# Management of Tongue-Tie Using Conventional Technique for Speech Clarity and Correcting Malocclusion: A Case Report

**DOI:** 10.7759/cureus.63756

**Published:** 2024-07-03

**Authors:** Aishwarya C Sawai, Monica Mahajani, Chitrika Subhadarsanee, Kuldeep Patil, Prasad V Dhadse

**Affiliations:** 1 Department of Periodontics and Implantology, Dr. Hedgewar Smruti Rugna Seva Mandal's Dental College and Hospital, Hingoli, IND; 2 Department of Periodontics and Implantology, Sharad Pawar Dental College and Hospital, Datta Meghe Institute of Higher Education and Research, Wardha, IND

**Keywords:** tongue-tie, conventional method, speech clarity, lingual frenum, ankyloglossia

## Abstract

Ankyloglossia, which is a congenital condition also referred to as tongue-tie, is described as a small lingual frenum that restricts tongue movement and its function. The main purpose of the frenum is to restrict the movement of the cheek, lip muscles, and tongue throughout the development of fetuses and maintain equilibrium between the developing bones, lip musculature, and tongue. The constriction of the buccal musculature counteracts the outward pressure that the tongue applies to the teeth. Arch width maintenance requires a state of equilibrium between these two muscle groups. Therefore, altering tongue position might additionally have an impact on a mandible's position. A 20-year-old female patient presented to the Periodontics Department with moderate ankyloglossia (Kotlow Class II). For the correction of the tongue-tie, conventional surgery with sutures was scheduled under local anesthesia. One week, one month, and three months follow-up, the patient demonstrated good healing. There was an improvement in speech clarity and tongue movements.

## Introduction

From the Greek words, which are agkilos meaning curved and glossa meaning tongue, the ankyloglossia word was derived [1]. Ankyloglossia also known by the name tongue-tie is a condition that is congenital and described by a short lingual frenulum which limits the movement of the tongue and negatively impacts the function [2,3]. The mandibular midline frenum, maxillary midline frenum, lower and upper buccal frenum on right and left, and the lingual frenum are among the frenum found in the oral cavity [4]. One of the only organs in the body that has one terminal attached and the other free is the tongue muscle. About 0.1%-10.7% of the population is affected by ankyloglossia. The female-to-male ratio for ankyloglossia is 1:2.5 [1]. The frenum can be positioned at or near the tip of the tongue and held tightly against the margins of the gingiva in the lower anterior teeth. There is a correlation between frenal pull and excessive muscle attachment and gingival tissue recession [5]. The buccal musculature constricts against the tongue's outward pressure on the teeth. Maintaining arch widths requires an equilibrium between these two muscle groups. Altered tongue position can affect mandible position as the tongue cannot be lifted upward, hence resulting in tongue thrust and open bite [4]. Ankyloglossia, characterized by restricted tongue mobility, can lead to feeding difficulties, speech issues, and malocclusion [5,6].

Assessment of the lingual frenum was done which was based on measurement using Kotlow's classification. The length of the tongue from the lingual frenum insertion into the base of the tongue to the tongue tip is described as free tongue [7]. Class I is mild ankyloglossia (12-16 mm), class II is moderate ankyloglossia (7-12 mm), class III is severe ankyloglossia (3-7 mm), and class IV is complete ankyloglossia (<3 mm).

## Case presentation

**Figure 1:**
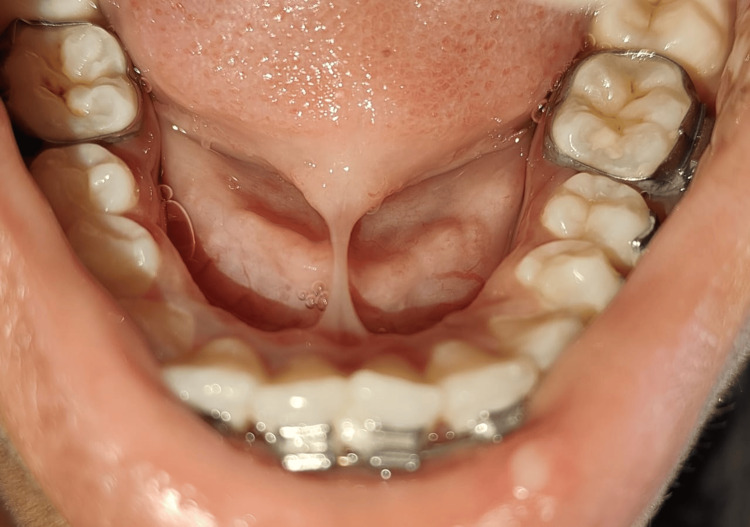
Restricted tongue movement before surgery

From the Department of Orthodontics, a 20-year-old female patient was reported to the Periodontics Department with high lingual frenum attachment which was restricting the tongue movement and leading to malocclusion. The subject gives no significant medical history and no relevant dental history. During the extraoral examination, no gross asymmetry was observed, and there was bilaterally smooth and synchronized temporomandibular joint movement. The submental and submandibular lymph nodes on both sides were inspected, which were neither tender nor palpable. An intra-oral examination revealed that the patient had a high lingual frenal attachment (Figure 1).

**Figure 2:**
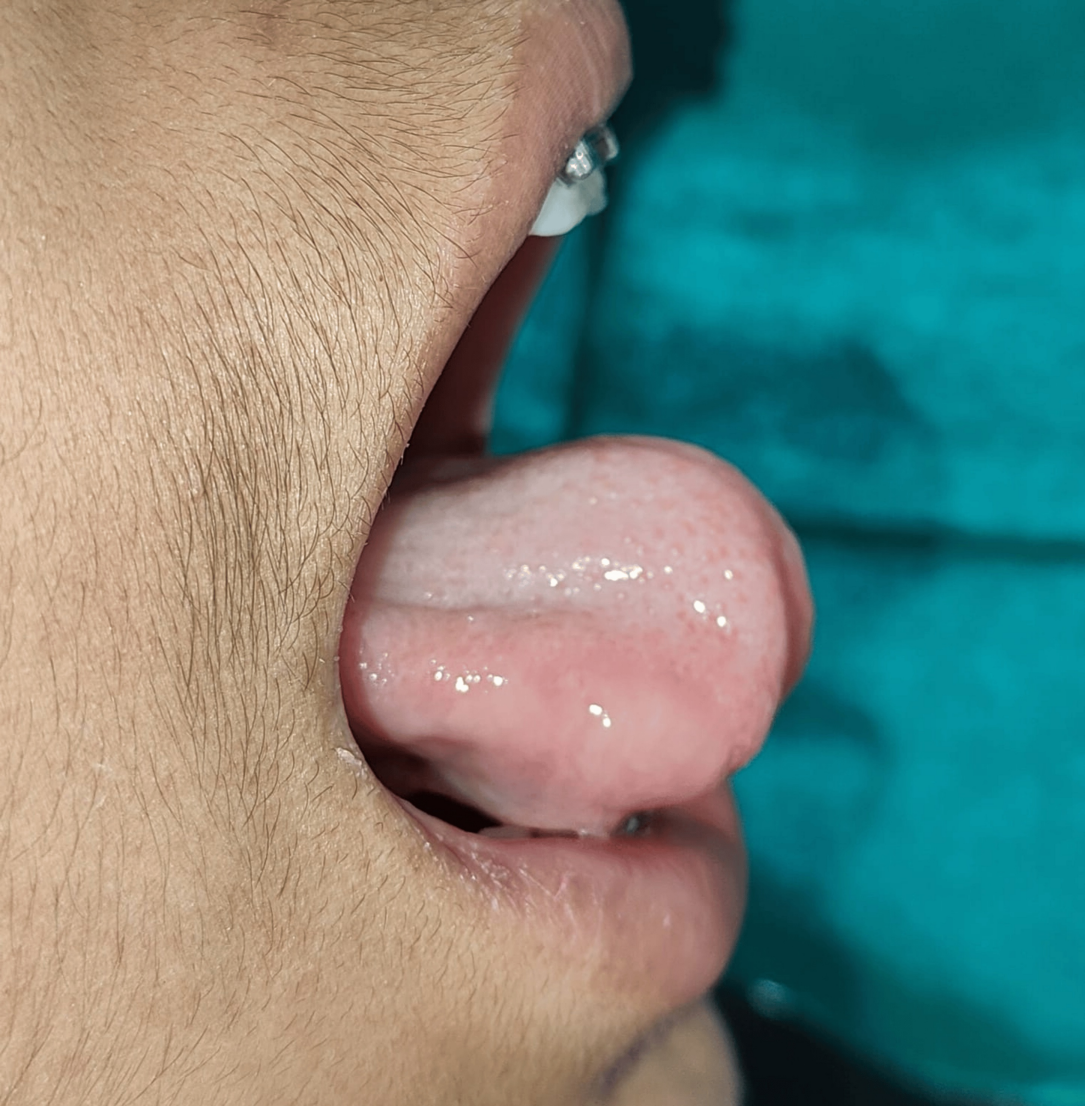
Limited outward extension of tongue

The patient was not able to pronounce letters such as “s, t, d, j, n, ch, th, zh”. Also, the patient was unable to place the tongue on the cingulum of the maxillary incisor and was unable to project the tongue tip outside the mouth (Figure 2).

**Figure 3:**
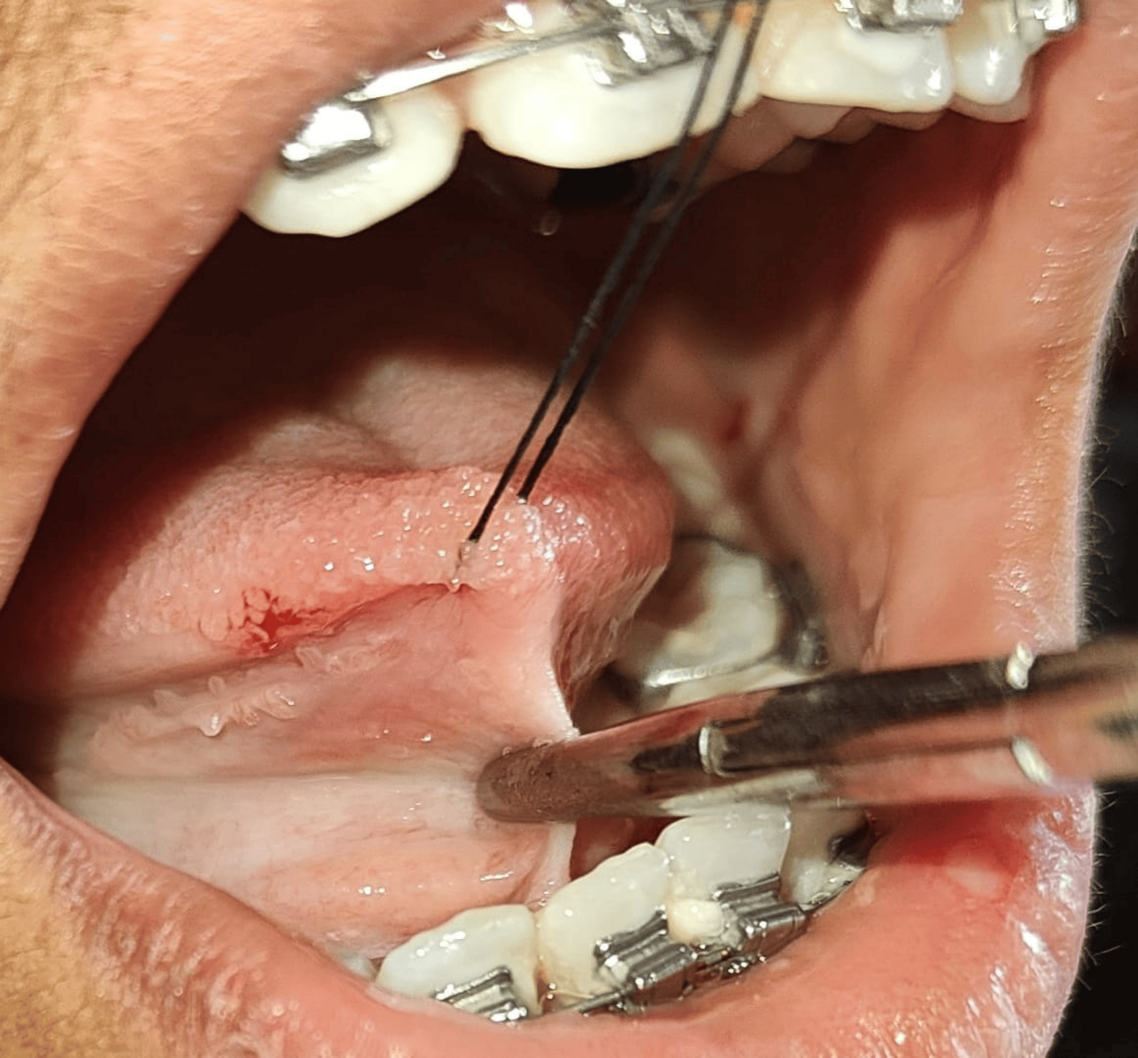
Hemostat holding the lingual frenum at the vestibule's depth

After initial oral prophylaxis (ultrasonic scaling), she was referred to the Oral Pathology Department for blood tests, including, clotting time (CT), bleeding time (BT), and hemoglobin (Hb). The patient was then recalled after seven days for a lingual frenectomy. Under all aseptic conditions and precautions, local anesthesia was given. The case was performed with a scalpel and 15 no-blade. Firstly, a hemostat was used to hold the lingual frenum at the vestibule's depth and the tongue tip was held with the help of a suture for the immobilization of the tongue (Figure 3).

**Figure 4:**
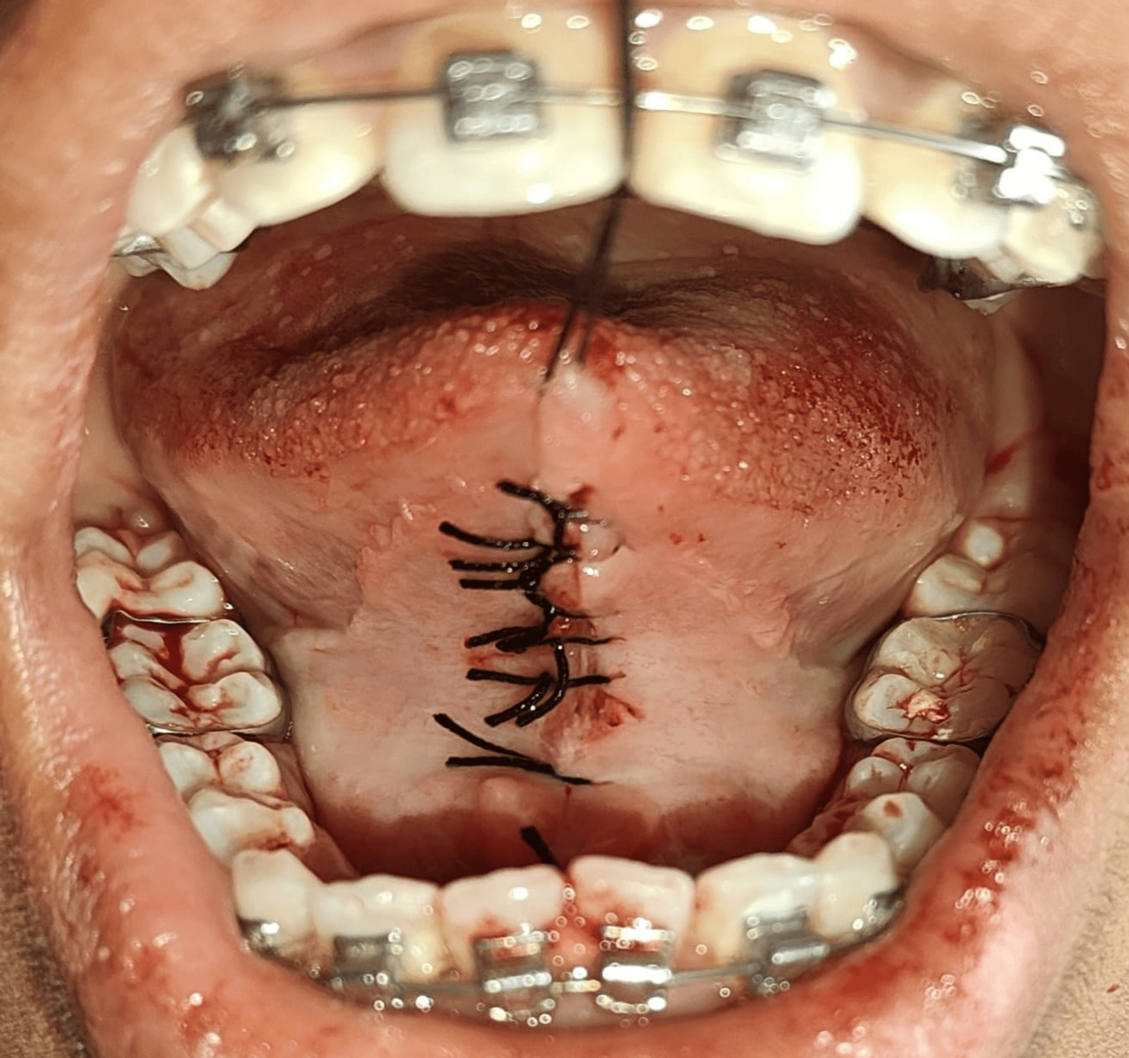
Hemostasis was attained using 4-0 mersilk sutures

A blade was then used to make two incisions on the hemostat's superior and inferior aspects, creating a triangle of tissue that was held in place by the hemostats and entirely eliminated. Blunt dissection was done, and any remaining fibers were removed. Hemostasis was attained by approximating the wound margins using 4-0 mersilk sutures (Ethicon, Bridgewater, NJ) (Figure 4).

**Figure 5:**
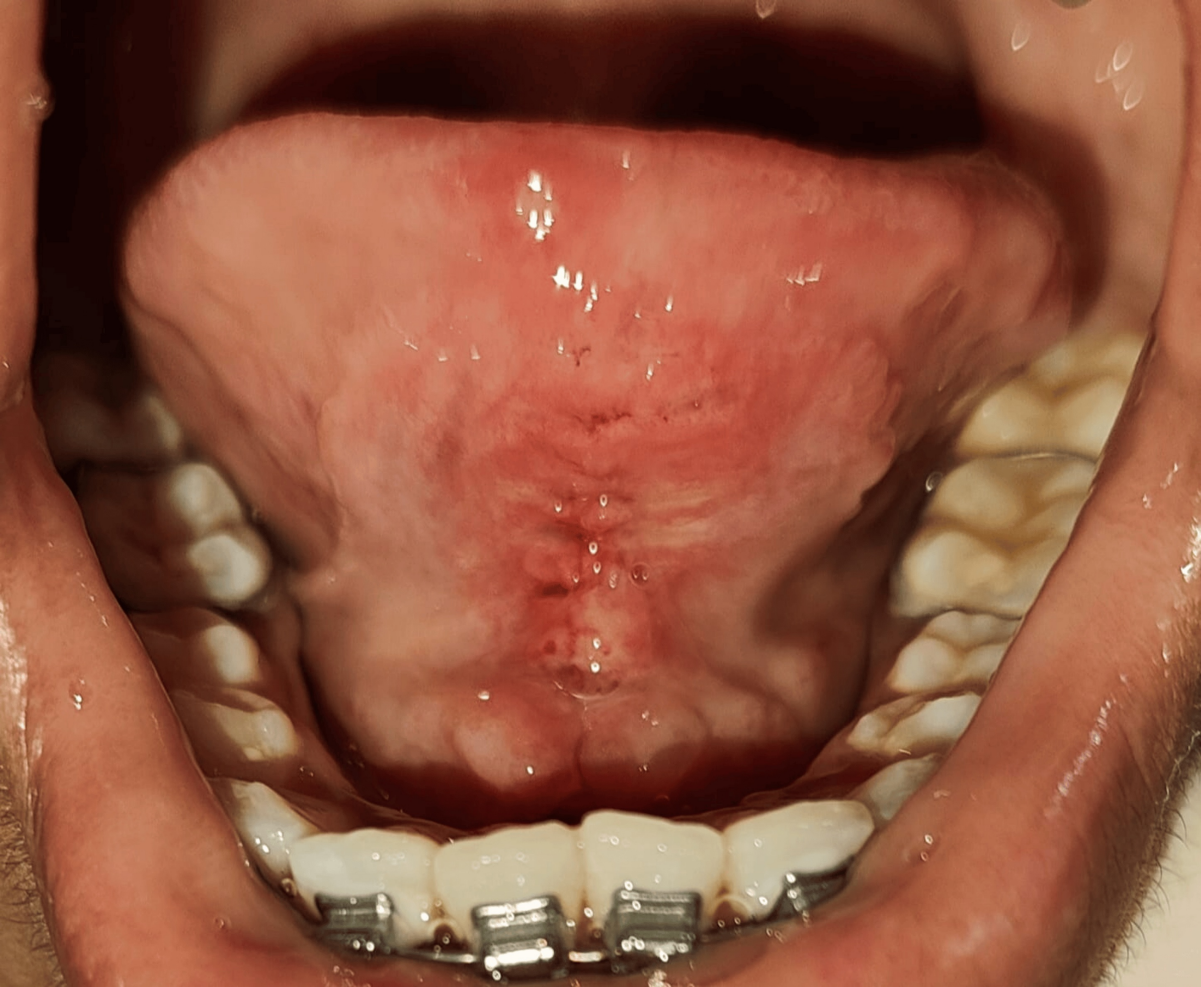
One-week post-operative view after suture removal

Analgesics and antibiotics were prescribed. At the one-week follow-up, suture removal was done, and the patient gave no history of postoperative bleeding and no discomfort present (Figure 5).

The patient was advised to do tongue exercises in front of the mirror after one week. The mentioned exercises were repeated for at least 10 to 20 times, two to three times a day [8].

Step 1

As much as possible, move the tongue's apex forward and in front of the nose tip, down to the chin tip, and then laterally at both the labial commissures.

Step 2

Make circular motions with the tongue in both counterclockwise and clockwise orientations and inside and outside the lips.

Step 3

Extend the tongue until it forms a pointed shape. 

Step 4

Ask the patient to touch the papilla with the help of the tongue tip. Touch the dorsum of the tongue to the palate. Suck the air with the help of the palate and tongue to generate a vacuum and improve adherence. Gradually expand your mouth and stretch the frenulum (the frenulum should be constricted; examine this position by looking into the mirror). Take the tongue away from the palate, resulting in an explosive sound. 

Step 5

Slowly open the mouth, then touch the papilla with the tongue tip, gradually increasing the speed.

Step-6

Position the tongue tip at the papilla, then move it forward and backward on the palate until it reaches the uvula.

**Figure 6:**
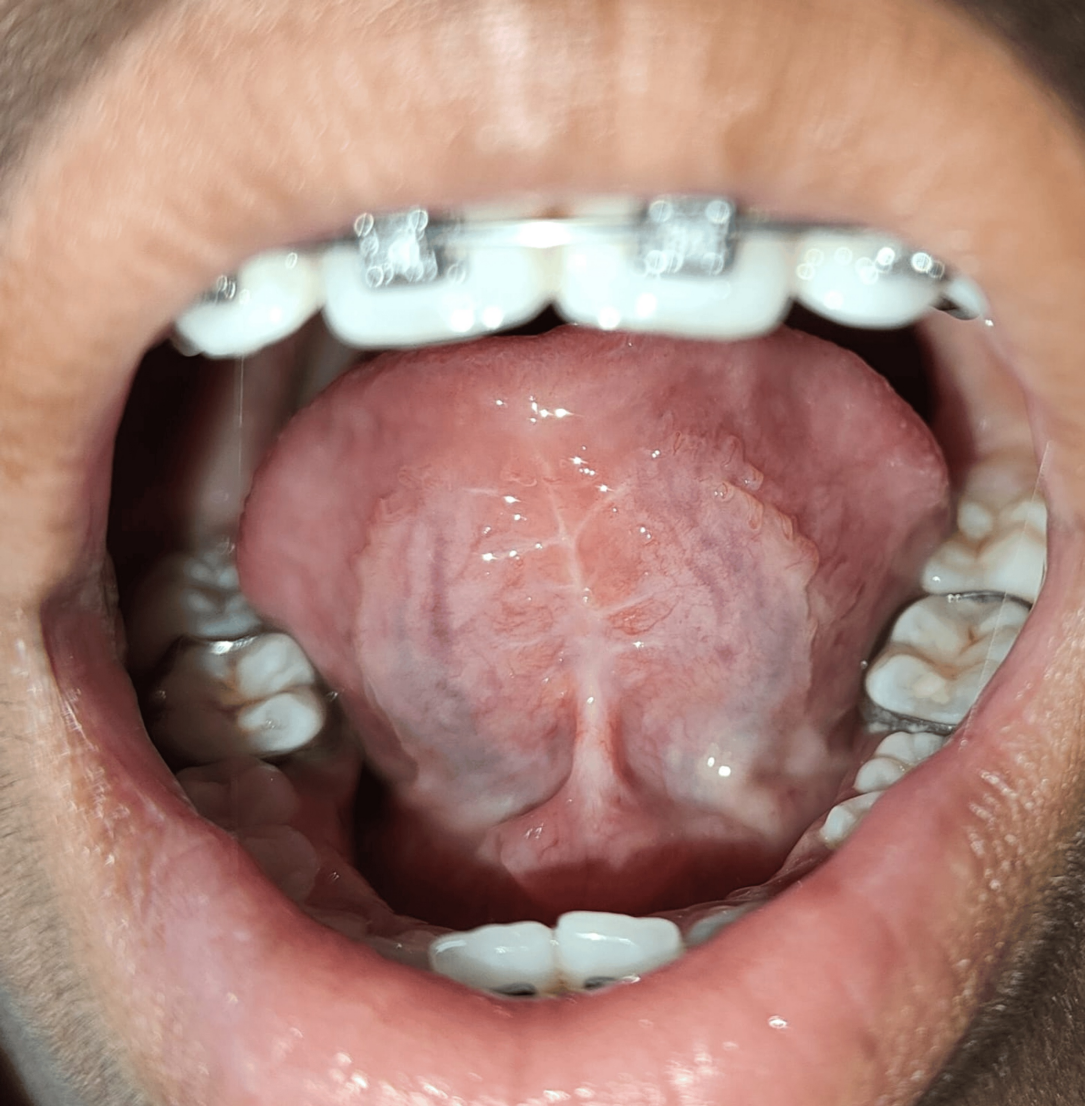
Follow-up recall examination after one month

**Figure 7:**
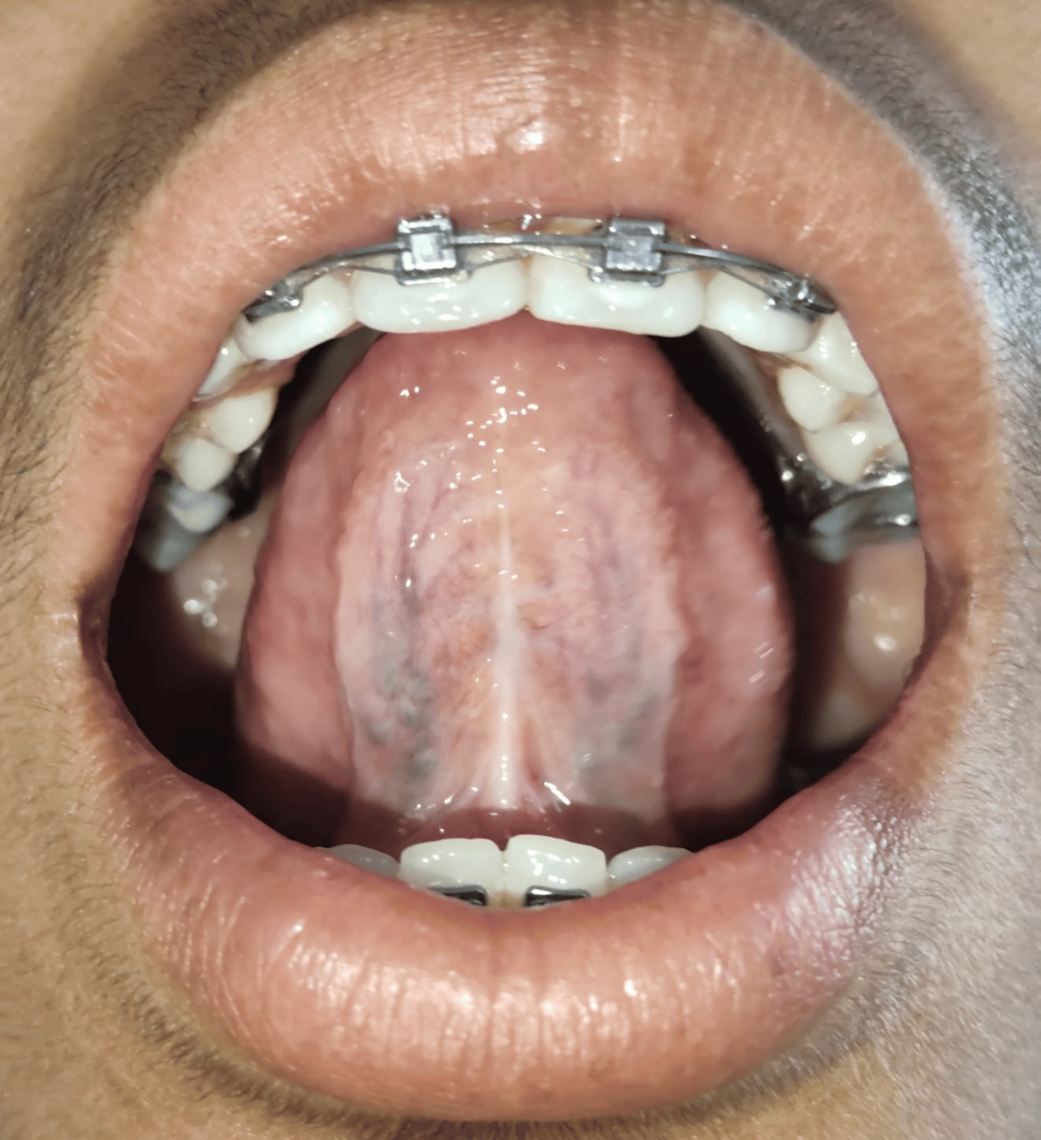
Follow-up recall examination after three months reveals tongue tip touching incisive papilla

**Figure 8:**
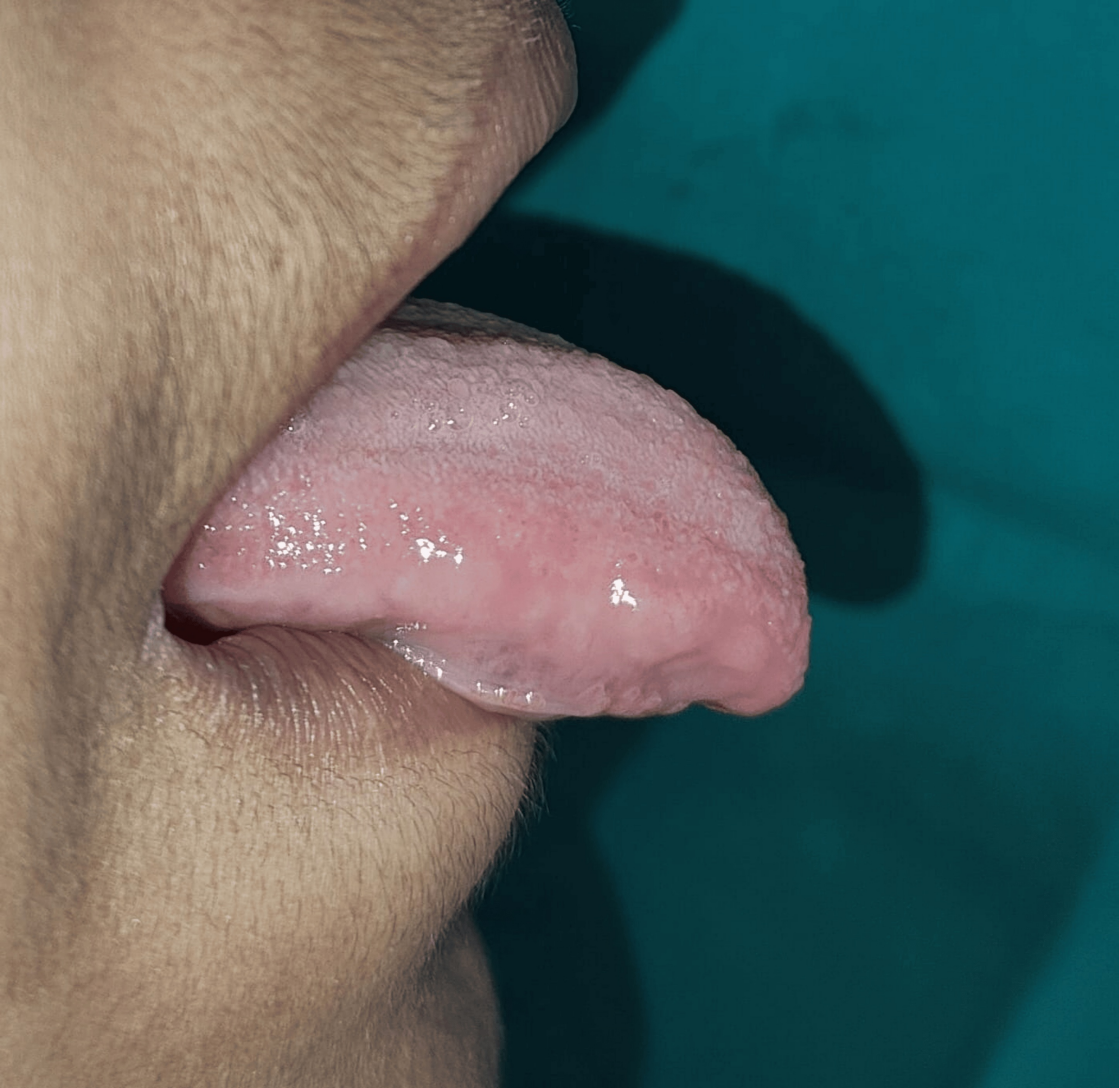
Follow-up recall examination after three months reveals no restricted tongue movement

A follow-up appointment was scheduled after one month (Figure 6) and three months (Figures 7, 8) postoperatively.

During one-month and three-month follow-ups, patients were asked to pronounce the letters “s, t, d, j, n, ch, th, zh” and an increase in the accuracy of the word pronunciations was seen, and adequate tongue movement and complete healing were observed upon an examination which gave the confidence to the patient and made this case expressive for speech clarity. The patient was then referred to the orthodontics department for further corrections of malocclusion and the main aim for treating ankyloglossia was to avoid relapse after orthodontic treatment which was achieved.

## Discussion

Frenum is introduced as a fold of muscle or tissue that joins, the jawbone to the tongue, cheeks, and lips. It is also known by the names: frenulum, frenula, and frena. A tongue tie is described as an overly small and lingual frenulum that is tight and limits the tongue motion. Specific mandibular discrepancy and speech impairments are caused in patients with ankyloglossia. Lingual dysfunction caused by short frenum is appreciated on the sagittal plane. Different individuals developed different types of malocclusions depending on the length of the frenum and the neuromuscular actions [9].

According to Kotlow’s assessment, ankyloglossia is classified into four classes, they are class I as mild, class II as moderate, class III as severe, and class IV as complete ankyloglossia [7]. Out of these, class III and class IV ankyloglossia should be given special consideration because it restricts tongue movement [10].

The literature mentions a number of suggested therapeutic approaches, including lasers, electrocautery, and surgical blades [11]. Various studies have also shown the release of tongue-tie as a safe and successful treatment with less or no complication. In order to achieve positive clinical outcomes, appropriate postoperative care, and general patient satisfaction, a thorough understanding of the sources of problems related to lingual frenectomy is required.

In conventional frenectomy, hemostats are used to guide the incisions and define the area that has to be delimited. Hemostats reduce the chance of unintentional soft tissue injury because the operator should only remove the tissue entirely by following the hemostats with a blade. Thus, clinicians with not much experience conducting frenectomy may feel more confident utilizing hemostats due to predetermined incision locations. At the same time when the frenulum is too short, the hemostat guides the incision line which lies close to the ventral surface of the tongue [12]. Also, the scalpel and blade technique are more cost-effective than laser and electrocautery; hence, these are some of the advantages of surgical technique. Surgical frenectomy methods have numerous disadvantages or risks, such as bleeding, infection at the operated site, damage to the salivary ducts nearby, pain, discomfort, swelling, and scar tissue formation.

Electrocautery offers varied advantages such as less time consumption, proper hemostasis, reduced operator fatigue, and suturing is not required. But at the same time, it has various disadvantages such as unpleasant odor, necrosis of tissue damage to adjacent structures due to generated heat [13].

Laser frenectomy is trending nowadays for multitudes of reasons such as less local anesthetic requirement, concurrent tissue cauterization, proper hemostasis, improved visualization, even depth of incision, and shorter operative time. However, some tissue studies show that when compared with laser and electrocautery, the scalpel technique shows less tissue injury, inflammation, and thermal damage [14]. Also, laser therapy is more expensive than electrosurgical and conventional techniques [5].

## Conclusions

The tongue is a highly vascular and dynamic structure. According to the grade of lingual adhesion, ankyloglossia, which can cause oral concerns such as limiting the tongue's range of motion and reducing its ability to perform functions such as speaking, tooth position, and swallowing, can be readily corrected via lingual frenectomy. The above-presented case of ankyloglossia was treated using a scalpel and blade, which resulted in favorable treatment outcomes and patient satisfaction with improved tongue movements, correction of a mandibular discrepancy, and speech problems.
